# Diplopia and extreme hyperamylasemia (>100,000 U/L) heralding aggressive IgG kappa myeloma: A case report and literature review

**DOI:** 10.1097/MD.0000000000048607

**Published:** 2026-05-08

**Authors:** Xiaohui Zhang, Weidong Zhou, Renhua Fan

**Affiliations:** a Department of Endocrinology, Changsha Hospital of Traditional Chinese Medicine (Changsha No. 8 Hospital), Changsha, China; b Department of Laboratory Medicine, Changsha Hospital of Traditional Chinese Medicine (Changsha No. 8 Hospital), Changsha, China.

**Keywords:** diplopia, hyperamylasemia, multiple myeloma, salivary-type amylase

## Abstract

**Rationale:**

While the production of amylase by epithelial tumors has been well-documented, its association with multiple myeloma (MM) is exceedingly rare, with only a limited number of cases reported. Moreover, to our knowledge, the presentation of binocular diplopia as a prominent and primary clinical feature in patients with MM and concurrent hyperamylasemia has not been widely reported.

**Patient concerns:**

This case report describes a 75-year-old man with immunoglobulin G kappa MM presenting with diplopia and extreme hyperamylasemia (>100,000 U/L) with normal lipase levels, a combination suggesting aggressive disease with extramedullary involvement.

**Diagnoses:**

Immunoglobulin G kappa-type MM and multiple organ dysfunction syndrome.

**Interventions:**

The patient received the first cycle of the VD regimen, consisting of bortezomib (2.2 mg, intravenous infusion, once weekly) and dexamethasone (10 mg, intravenous drip, days 1 and 2).

**Outcomes:**

Despite aggressive treatment, the patient’s condition deteriorated rapidly and ultimately succumbed to the disease in the early morning of the fourth week post-diagnosis.

**Lessons:**

Patients with amylase-producing myeloma exhibit larger tumor burdens, more extensive extramedullary dissemination, and significantly shorter survival than typical MM cases. Binocular diplopia may indicate skull base extramedullary involvement. Unexplained hyperamylasemia with normal lipase should prompt malignancy screening. Early diagnosis is crucial given the rapidly fatal course.

## 1. Introduction

Multiple myeloma (MM) is a malignant neoplastic disease originating from terminally differentiated B cells, characterized by the malignant proliferation of plasma cells and the excessive secretion of monoclonal immunoglobulins or their fragments (M proteins). It is one of the most common hematological malignancies.^[1]^ The clinical symptoms of MM are highly variable, with patients often presenting with bone pain, fractures, anemia, renal insufficiency, hypercalcemia, and recurrent infections. In cases of extramedullary infiltration, patients also develop nonspecific symptoms such as nausea, vomiting, abdominal pain, diarrhea, paraplegia, and motor or sensory disturbances.^[2,3]^ To date, no reports in the literature, either domestically or internationally, have documented a case of MM diagnosed due to the primary symptom of binocular diplopia.

Amylase measurement is a commonly used laboratory test, with abnormal elevations primarily aiding in the differential diagnosis of pancreatic and salivary gland disorders. When clinical presentations and imaging exclude diseases of the pancreas and salivary glands, other organ or systemic diseases must be considered. Hyperamylasemia associated with malignancies is relatively rare, and its concurrence with MM is even more uncommon. Several studies have reported that patients with elevated amylase levels in the context of malignancy tend to have higher tumor burdens, more rapid disease progression, and poorer prognoses.

This case report presents the diagnostic and therapeutic process of a patient whose primary clinical manifestations were binocular diplopia and hyperamylasemia. Through the comprehensive analysis of this case and a review of the relevant literature, we summarize the diagnostic workup, therapeutic strategies, and prognostic outcomes of MM associated with hyperamylasemia. This report aims to provide insights and practical experience for the diagnosis and treatment of MM in patients with hyperamylasemia.

## 2. Case report

A 75-year-old male patient presented to the outpatient clinic in Changsha Hospital of Traditional Chinese Medicine on June 19, 2024, with complaints of fatigue, poor appetite, and abnormal weight loss persisting for 6 months. Laboratory tests revealed the following: complete blood count: hemoglobin 92.00 g/L (low) and platelets 78.00 × 10^9^/L (low); biochemical panel: serum albumin 32.20 g/L (low), globulin 83.80 g/L (high, reference range: 20–30 g/L), albumin/globulin ratio 0.38 (low), fasting blood glucose 6.49 mmol/L (high), glycated hemoglobin (HbA1c) 8.90% (high), and calcium 2.67 mmol/L (high); urinary results: urinary β_2_-microglobulin 6.15 mg/L (high, reference range: <0.3 mg/L), urinary protein quantification 620.00 mg/L (high, reference range: <150 mg/L), and urine albumin/creatinine ratio 666.67 mg/g (high, reference range: <30 mg/g). These findings suggested anemia, thrombocytopenia, abnormally elevated globulin levels, an inverted albumin-to-globulin ratio, elevated urinary protein, hypercalcemia, and hyperglycemia. Hospital admission was recommended for further investigation, but the patient and his family declined and opted for home observation.

On June 26, 2024, the patient presented to a local hospital with sudden-onset dizziness and diplopia lasting 5 days. Imaging studies, including head magnetic resonance imaging, diffusion-weighted imaging, and magnetic resonance angiography, revealed a bone destruction of the clivus with associated soft tissue mass formation, brain atrophy, cerebral arterial sclerosis with stenosis, suggestive of neoplastic lesion, chordoma, or other malignancies. The patient was subsequently admitted to Changsha Traditional Chinese Medicine Hospital on the same day for further diagnostic evaluation.

The patient had a medical history of diabetes, hypertension, and psoriasis. The patient’s medications included subcutaneous insulin and irbesartan for glycemic and hypertension control, respectively. Psoriasis was well-controlled without medication. He denied alcohol use or recent infections, excluding these common causes of hyperamylasemia. He had been a smoker for over 40 years and had 3 children (2 daughters and 1 son), all in good health. There was no family history of hereditary diseases. The physical examination includes: first, vital signs: body temperature 36.5°C, respiratory rate 18 breaths/min, pulse rate 68 beats/min, blood pressure 125/65 mm Hg, height 170 cm, weight 66 kg, and body mass index 22.8 kg/m^2^; second, general appearance: normally and well developed, cooperative in the examination, with moderate mental status; third, skin findings: no palpable superficial lymphadenopathy, no significant jaundice on the skin and sclera, and no signs of palmar erythema or spider angiomas. Multiple irregularly bordered, well-demarcated, dark purple plaques of varying sizes were observed on the limbs and back, the largest measuring approximately 6 × 10 cm on the back and the smallest measuring 2 × 3 cm on the lateral side of the left calf. These plaques were non-confluent, elevated, and covered with silvery, shell-like scales. The surrounding skin was normal, with no signs of redness, swelling, exudation, or tenderness; fourth, respiratory system: clear breath sounds bilaterally with no audible dry or wet rales; fifth, cardiovascular system: normal heart size, regular rhythm, no murmurs; sixth, abdomen: soft and non-tender, with no rebound tenderness or organomegaly. There is no tenderness in the hepatic or renal regions; seventh, other examinations showed no edema in extremities, no main abnormalities in neurological examination. No redness, swelling, or tenderness was observed in the parotid glands bilaterally; eighth, specialized examination on ophthalmology: a marked restriction in lateral movement in right eye, with no limitation in upward, downward, or medial movements. There is no restriction in ocular movements in left eye. Diplopia was pronounced when looking to the right, upper right, and lower right, with clear horizontal separation of images. Diplopia was less pronounced in the upper and lower left visual fields.

Upon hospital admission, the laboratory tests revealed the following: first, rheumatoid factor, anti-streptolysin O, antinuclear antibody profile, and parathyroid hormone were all negative; second, complete blood count: hemoglobin 82.00 g/L (low) and platelets 80.00 × 10^9^/L (low); third, biochemical tests: uric acid 773.00 µmol/L (high), calcium 2.87 mmol/L (high), lactate dehydrogenase 411.00 U/L (high), albumin 28.00 g/L (low), globulin 79.50 g/L (high), and albumin-to-globulin ratio 0.35 (low); fourth, amylase and lipase: blood amylase 145,295.00 U/L (significantly high, normal range: 35–135 U/L), serum lipase 59.90 U/L (normal range: 1–60 U/L), and urine amylase 132,891.00 U/L (significantly high); fifth, amylase-to-creatinine clearance ratio (*C*_am_/*C*_cr_): 1.9% (normal range: 2–5%, <1% indicates macroamylasemia, calculated by [urinary amylase concentration/blood amylase concentration] × [serum creatinine concentration/urinary creatinine concentration] × 100%).^[4]^ Based on the patient’s elevated urine amylase, *C*_am_/*C*_cr_, and results of polyethylene glycol precipitation (normal value <52%),^[5]^ macroamylasemia was excluded. Further isoenzyme analysis of serum amylase was conducted at the Laboratory Medicine Department of the Second Xiangya Hospital of Central South University, yielding the following results: total amylase: 176,030.0 U/L (normal range: 35.0–135.0 U/L); pancreatic-type amylase (P): 2769.0 U/L (normal range: 8.0–53.0 U/L); salivary-type amylase (S): 173,261.0 U/L. These findings suggested that the elevated amylase was primarily salivary-type (S), ruling out pancreatitis as the cause of hyperamylasemia. Parotid gland ultrasonography showed no abnormalities, excluding parotitis as a potential cause of the elevated amylase levels.

**Figure 1.**
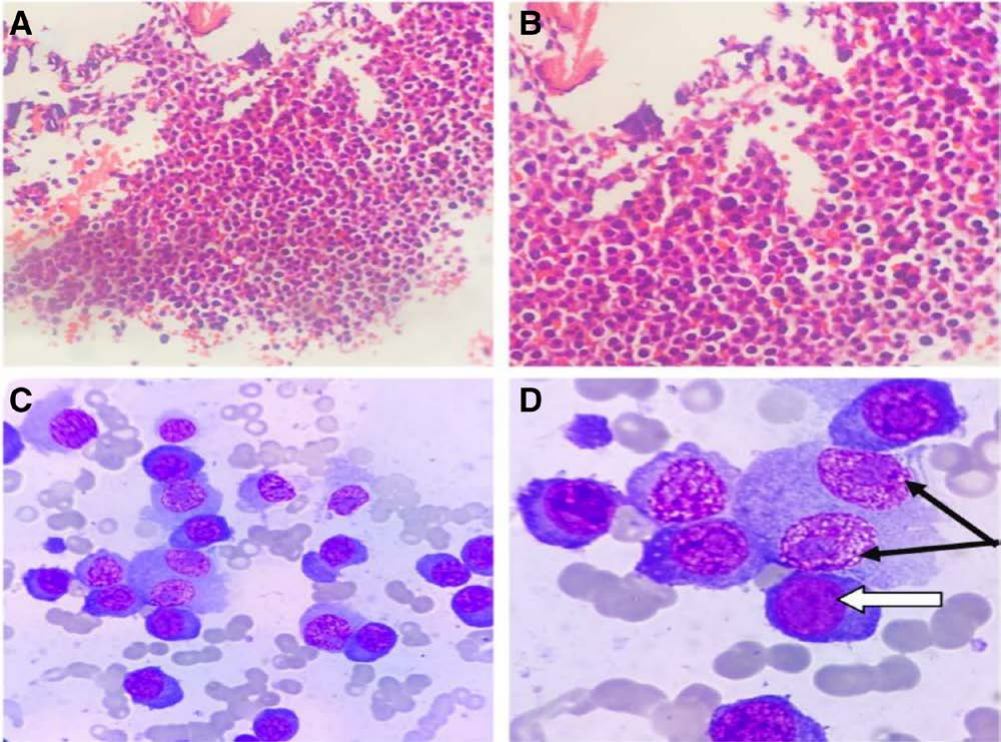
Bone marrow histopathology and cytology. (A and B) Bone marrow histopathology showing extensive plasma cell proliferation under microscopy. (C and D) Bone marrow cytology, with black arrows indicating binucleated malignant plasma cells and white arrows indicating eccentric nuclei.

**Figure 2.**
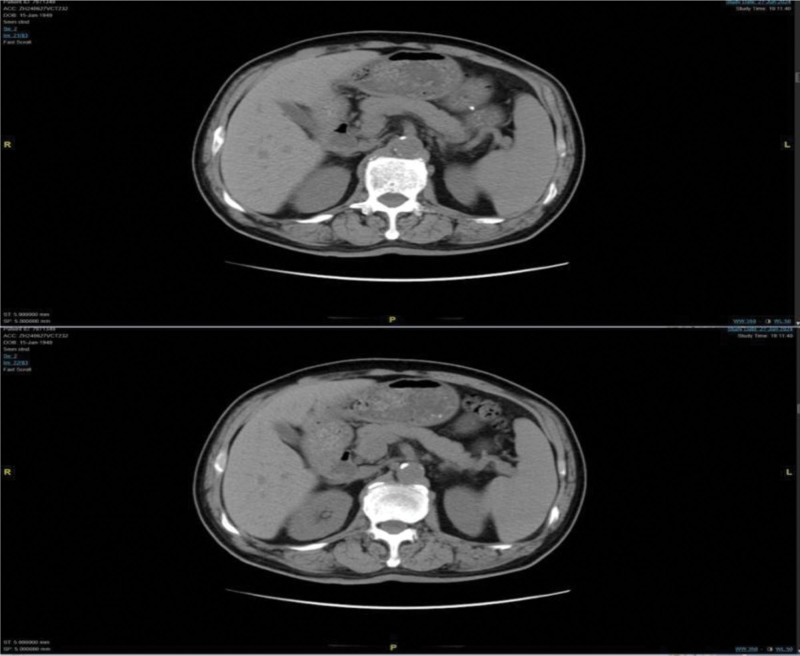
Abdominal CT showing no inflammatory or pathological changes in the pancreas. CT = computed tomography.

**Figure 3.**
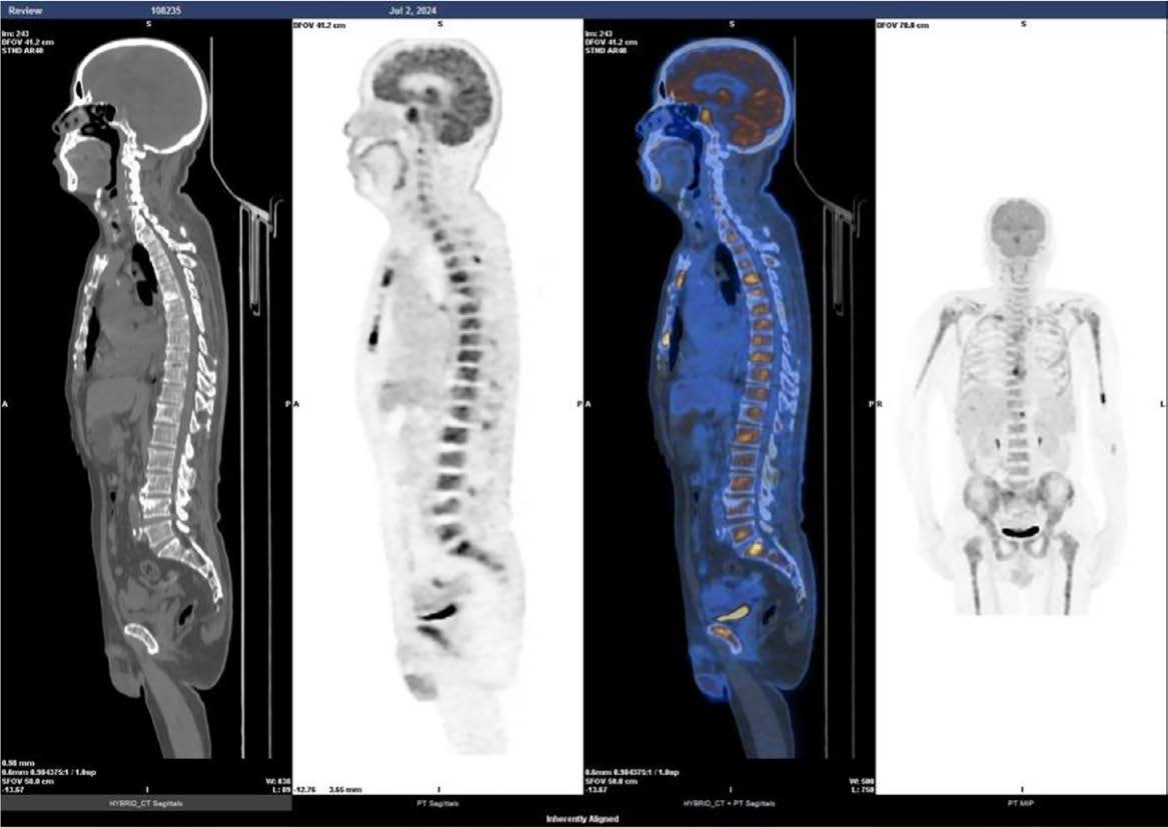
Whole body PET-CT revealing multiple areas of radiotracer uptake, indicating extensive bone destruction. PET-CT = positron emission tomography–computed tomography.

Further detailed diagnostic immunological, hematological, histological, and radiological evaluations were also performed in the patient. First, immunological markers: immunoglobulin G (IgG) 82.000 g/L (high), immunoglobulin M 0.196 g/L (low), immunoglobulin A (IgA) 0.206 g/L (low), kappa light chain 7140.00 mg/dL (high), lambda light chain <30.0 mg/dL (low), and β_2_-microglobulin 10.92 mg/L (high); second, serum protein electrophoresis: gamma-globulin 52.3% (high), M protein percentage 49.9%, and serum M protein concentration 57.16 g/L; third, serum immunofixation electrophoresis: SP (+), IgG (+), IgA (−), immunoglobulin M (−), κ (+), λ (−); fourth, bone marrow findings (Fig. 1C and D): blast plasma cells 47.50% (high) and immature plasma cells 48.50% (high); fifth, flow cytometry immunophenotyping: monoclonal plasma cells accounted for approximately 61.55% of nucleated cells. Immunophenotyping revealed CD38+, CD138+, CD19−, CD20−, CD28+, CD56+, CD81−, CD200+ (low expression), with intracellular immunoglobulin kappa light chain restriction, consistent with monoclonal plasma cells; sixth, bone biopsy results (Fig. 1A and B): histological examination showed marked plasma cell proliferation, consistent with plasma cell proliferative disorders, pending clinical correlation. Immunohistochemistry results showed CD20 (−), CD79α (+), CD3 (−), CD21 (−), CD56 (+), CKpan (−), TDT (−), Cyclin D1 (−), CD138 (+), CD38 (−), κ (+), λ (+), and Ki-67 (70%); seventh, abdominal computed tomography (CT) scan (Fig. 2) revealed normal pancreas appearance. Bilateral sacroiliitis was noted, with heterogeneous reduction in bone density of the sacral and coccygeal vertebrae and bilateral sacral bones, etiology undetermined; eighth, cranial base CT with 3D reconstruction showed abnormal density in the sphenoid sinus with absorption and destruction of the clivus. The whole-body positron emission tomography (PET)-CT (completed on July 2, 2024, at Hunan Cancer Hospital, Fig. 3) revealed multiple sites of bone destruction involving the skull, vertebrae, scapulae, sternum, humerus, radius, ribs, clavicles, pelvic bones, and femurs. Corresponding areas showed abnormal radioactive uptake on PET, strongly suggestive of malignancy, with a high likelihood of MM. The key diagnostic findings are summarized in Table 1.

**Table 1:** Key diagnostic findings.

Parameter	Finding	Reference range/significance
Serum amylase	145,295.00 U/L	35–135 U/L
Serum lipase	59.90 U/L	1–60 U/L (normal, rules out pancreatitis)
Amylase isoenzyme	S-type: 98.4%	Suggests salivary/non-pancreatic origin
Macroamylasemia	Excluded	*C*_am_/*C*_cr_: 1.9%; PEG precipitation test
Parotitis	Excluded	No clinical or US evidence
Bone marrow aspirate	Blast plasma cells 47.50% (high), immature plasma cells 48.50% (high)	Supports MM diagnosis
Serum immunofixation	IgG kappa M-protein	Confirms monoclonal gammopathy
PET-CT imaging	Extensive osteolytic lesions	Consistent with myelomatous involvement

*C*_am_/*C*_cr_ = amylase-to-creatinine clearance ratio, MM = multiple myeloma, PEG = polyethylene glycol, PET-CT = positron emission tomography–computed tomography, US = ultrasonography.

Based on the patient’s clinical presentation, laboratory findings, and imaging results, a diagnosis of “IgG kappa-type multiple myeloma” was established. Following consultation with oncology and hematology specialists, it was recommended that the patient be transferred to a specialized department for radiotherapy and chemotherapy. One week after admission, follow-up blood tests revealed hemoglobin levels of 71.00 g/L (low) and platelet count of 45.00 × 10^9^/L (low). Repeat serum amylase was 143,557.40 U/L (significantly high). However, the patient and their family declined transfer to the oncology and hematology department for radiotherapy and chemotherapy, opting instead for palliative care. The patient was discharged on July 8, 2024. On July 12, 2024, the patient presented to a local primary care hospital with worsening dizziness, headache, and binocular diplopia. Despite receiving symptomatic treatment, including nutritional support, there was no improvement in symptoms. Due to rapid disease progression, the patient was transferred to the “Hunan Cancer Hospital” on July 21, 2024, where serum amylase was measured at 58,798.99 U/L. On July 24, the patient received their first cycle of the VD regimen, consisting of bortezomib (2.2 mg, intravenous infusion, once weekly) and dexamethasone (10 mg, intravenous drip, days 1 and 2). The VD regimen was selected for its rapid efficacy against high-risk myeloma with extramedullary involvement, particularly given the patient’s poor performance status which contraindicated lenalidomide-based therapy due to myelosuppression and thromboembolism risks. Although local radiotherapy to the clival lesion was considered for diplopia control, rapid systemic deterioration precluded its implementation. Supportive care for metabolic parameters was maintained throughout treatment.

**Figure 4.**
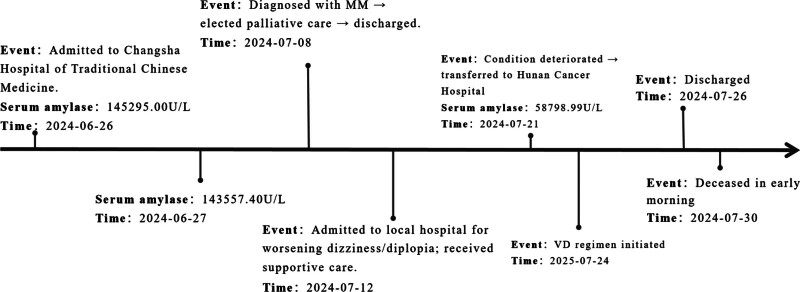
Clinical timeline showing key events and amylase trends. MM = multiple myeloma, VD = bortezomib and dexamethasone.

The patient was discharged on July 26, 2024. Despite treatment, the patient’s condition deteriorated rapidly, and passed away in the early morning of July 30, 2024, 4 weeks after the initial diagnosis. The clinical timeline of key events and amylase trends is presented in Figure 4.

*Family’s perspective*: The family expressed profound shock at the disease’s rapid progression. Their decision to pursue palliative care was made to avoid invasive interventions, and they hope this case raises awareness of myeloma’s atypical presentations for earlier future diagnosis.

## 3. Methods

We collected the medical records of the patient, which included clinical characteristics, laboratory parameters, pathological results, abdominal CT imaging, whole-body PET-CT imaging, treatment plans, and clinical outcomes. This case study was approved by the institutional review board of Changsha Traditional Chinese Medicine Hospital (Changsha No. 8 Hospital). Informed consent for publication was obtained from the patient’s family due to the patient’s demise. A comprehensive literature search was conducted in PubMed, Embase, Web of Science, and CNKI databases from January 1951 to March 2025. The search terms included “multiple myeloma,” “hyperamylasemia,” and “salivary amylase” along with their related keywords and Medical Subject Headings (MeSH) terms.

## 4. Discussion

MM with concurrent hyperamylasemia typically presents with clinical manifestations such as bone pain, anemia, renal dysfunction, hypercalcemia, and infections. When extramedullary infiltration occurs, nonspecific symptoms such as nausea, vomiting, abdominal pain, diarrhea, paraplegia, and motor or sensory impairments may also develop. The clinical presentation of MM with hyperamylasemia is complex, lacking specificity, and often leads to misdiagnosis as pancreatitis in cases presenting with abdominal pain and vomiting.^[6]^

In this case, the patient exhibited significantly elevated serum amylase levels with normal lipase levels, without symptoms of abdominal pain or vomiting. Additionally, pancreatic CT showed no evidence of peripancreatic exudation or pancreatic enlargement, ruling out a diagnosis of pancreatitis. The absence of typical MM symptoms, such as bone pain or pathological fractures, delayed initial suspicion of MM. Apart from nonspecific symptoms of malignancy, including dizziness, fatigue, poor appetite, and anemia, the patient also presented with binocular diplopia. To the best of our knowledge, “binocular diplopia” as a prominent and primary clinical feature in the context of MM with hyperamylasemia has not been widely reported. The probable mechanism for diplopia in this patient was extramedullary infiltration of MM into the skull, with cranial bone destruction involving the right abducens nerve, resulting in diplopia. Hyperamylasemia was a key diagnostic breakthrough in identifying MM in this case, while diplopia represented a clinical manifestation of extramedullary MM infiltration.

Amylase is commonly used in clinical practice for the diagnosis of pancreatic diseases and is found in various organs and tissues. In addition to the pancreas, amylase is secreted by the salivary glands, ovaries, fallopian tubes, lungs, testes, seminal fluid, and mammary glands.^[7,8]^ Amylase levels significantly increase in acute pancreatitis but can also fluctuate in other conditions involving exocrine dysfunction. There are 2 major isoenzymes of amylase: the P isoenzyme (pancreatic-type) and the S isoenzyme (salivary-type). Elevations in P isoenzyme are primarily associated with pancreatic disorders, intestinal obstruction, intestinal perforation, renal failure, or postoperative states, while elevations in S isoenzyme can occur in conditions such as mumps, anorexia nervosa, bulimia, ectopic pregnancy, salpingitis, chronic alcoholism, ovarian cysts, papillary serous ovarian carcinoma, lung cancer, colorectal cancer, MM, renal failure, or postoperative states.^[7]^ In this patient, there was no redness, swelling, warmth, or pain in the bilateral parotid regions, and parotid gland ultrasound showed no abnormalities, ruling out mumps. Isoenzyme analysis of serum amylase revealed a predominance of salivary-type (S) amylase, consistent with findings reported in the previous literatures.^[9–14]^ A minor fraction of pancreatic-type (P) amylase (1.6%) was also detected, suggesting that ectopic amylase production may involve predominantly S-type secretion with minimal P-type secretion. Given the repeated abnormal elevation of serum amylase in this patient, a peripheral blood smear was performed by laboratory physicians on the second day after admission in the hospital. The smear revealed numerous abnormal plasma cell-like cells and rouleaux formation of red blood cells, consistent with previous findings reported by Guo et al.^[15]^ Together with abnormal serum globulin levels and an inverted albumin-to-globulin ratio, the findings strongly suggested the possibility of MM. Subsequent confirmatory tests, including serum protein electrophoresis, immunofixation electrophoresis, bone marrow examination, and flow cytometry immunophenotyping, established the diagnosis of MM. In most reported cases, MM is diagnosed first, followed by the incidental finding of elevated amylase. However, in this patient, the breakthrough leading to the diagnosis of MM was the abnormal elevation of serum amylase, underscoring the need for increased awareness of tumor-associated hyperamylasemia among clinicians. In patients with MM and elevated circulating immunoglobulins, macroamylasemia should be considered as a potential cause of hyperamylasemia. Macroamylase results from the binding of amylase to proteins or immunoglobulins, forming high molecular weight complexes that cannot be cleared by the kidneys, leading to elevated serum amylase levels.^[16]^ However, in this patient, both serum and urinary amylase levels were consistently elevated. Following polyethylene glycol precipitation, amylase activity was measured at <52%, and the *C*_am_/*C*_cr_ was 1.9% (>1%), effectively ruling out macroamylasemia.

Hyperamylasemia caused by ectopic amylase production in tumor tissues was first reported by Weiss et al in 1951,^[17]^ with tissue culture and immunohistochemical techniques later confirming amylase production by cancer cells. Subsequently, hyperamylasemia has been described in numerous epithelial malignancies,^[6,18–20]^ particularly lung and ovarian cancers, as well as malignancies of the breast, uterus, and stomach. In recent years, elevated amylase levels have also been observed in non-epithelial malignancies, including MM.^[18,21]^ The first report of hyperamylasemia due to ectopic amylase production in MM was published in 1988, followed by additional case reports of amylase-producing MM.^[22–28]^ Table 2 provides a summary of the reported cases, showing a median age of 65 years at diagnosis, with a male-to-female ratio of 1.3:1. The primary clinical manifestations included abdominal pain, vomiting, and back pain. The mean amylase level across cases, including the present case, was 15,792.39 U/L, with a minimum value of 1153 U/L. Regarding amylase classification, 2 cases were identified as macroamylasemia, while most cases involved the S isoenzyme (salivary-type). The predominant myeloma subtype was IgA, followed by IgG. In the light chain, the ratio of λ:к is 1:1. Most cases were found in Japan, with an average survival time of 26 months. The shortest survival time, 4 weeks, was observed in this case. Almost all reported cases exhibited extramedullary metastasis. Fujii et al^[12]^ managed a case of MM with markedly elevated serum amylase and extensive extramedullary dissemination, and they reviewed 5 additional cases (including their own) of MM with significant hyperamylasemia. All cases involved widespread extramedullary dissemination, with extramedullary tumors and/or myelomatous pleural effusion or ascites. Among these, 3 cases showed extensive bone destruction, and 4 patients had an initial survival period of less than 1 year. These findings suggest that amylase-producing MM is associated with a higher tumor burden, more rapid disease progression, poorer prognosis, and shorter survival time. The present case involves IgG kappa-type MM, with the highest reported serum amylase level to date. The patient exhibited widespread extramedullary dissemination, including skull and rib bone destruction, and had the shortest survival time (4 weeks) among reported cases. This further corroborates the conclusions of previous studies.

**Table 2:** Clinical features and survival time of patients with multiple myeloma (MM) and concurrent hyperamylasemia.

First author	Sex	Age	Clinical symptoms	Amylase (U/L, normal range: 35–135 U/L)	Amylase isoenzyme type	Myeloma subtype	Treatment regimen	Country	Year	Survival time (mo)	Extramedullary involvement
Machida et al	Female	72	–	2674	Macroamylasemia, unclassified	IgA-λ type	Melphalan, prednisone	Japan	2010	60	–
Lopeza et al	Female	72	Nausea, upper abdominal pain	3071	S type	λ light chains	Melphalan, prednisone	Spain	1993	58	–
Kanno et al	Male	71	Upper abdominal discomfort	31,070	S type	IgD-λ type	Vincristine, cyclophosphamide, melphalan, prednisone	Japan	1988	–	Yes
Guo et al	Male	65	Upper abdominal pain	2306	–	IgG-λ type	Bortezomib, thalidomide, dexamethasone (2 cycles); bortezomib, cyclophosphamide, dexamethasone (1 cycle)	China	2016	3	Yes
Nair et al	Female	47	Abdominal distension, vomiting	2306	–	–	Melphalan, prednisone	USA	1995	24	Yes
Ross et al	Female	42	Abdominal pain with nausea and mild vomiting	3142	–	κ light chains	Adriamycin, dexamethasone, and bone marrow transplantation	UK	1998	32	Yes
Pinelli et al	Male	79	Fatigue, weight loss, low back pain	3492	S type	λ light chains	Cyclophosphamide, prednisone	Italy	2003	16	Yes
Hata et al	Male	53	Upper abdominal pain	1600	S type	IgA-λ type	Cyclophosphamide, vincristine, prednisone	Japan	1985	18	Yes
Ohtsuki et al	Female	64	Low back pain	–	–	Nonsecretory type	Vincristine, cyclophosphamide, melphalan, prednisone; intrapleural methotrexate, cytarabine, hydrocortisone	Japan	1989	–	Yes
Takeya et al	Male	53	Upper abdominal pain	1600	S type	IgA-λ type	Melphalan, prednisone	Japan	1989	11	Yes
Moriyama et al	Male	70	–	2401	S type	IgD-λ type	–	Japan	1990	2	Yes
Sagristani et al	Male	54	Low back pain, fatigue	1153	Macroamylasemia, unclassified	IgA-κ type	Vincristine, idarubicin, dexamethasone	Italy	1998	2.80	–
Shigemura et al	Female	68	–	43,020	S type	IgG-λ type	Peripheral blood stem cell transplantation (PBSCT)	Japan	2003	64	–
Sosnoff et al	Male	61	Severe back pain	10,448	S type	κ light chains	Bortezomib, dexamethasone	USA	2010	6	Yes
Bloemenda et al	Female	71	Fever, abdominal pain	7567	S type	–	Melphalan, prednisone	Netherlands	1992	60	Yes
Moriyama et al	Female	85	–	1836	S type	IgA-λ type	–	Japan	1991	–	–
Moriyama et al	Male	70	–	1554	S type	IgA-κ type	–	Japan	1991	–	–
Li et al	Male	60	Low back and right lower limb pain	19,470	–	IgG-к type	Bortezomib, thalidomide, dexamethasone	China	2022	12	Yes
Current case	Male	75	Binocular diplopia, fatigue, poor appetite	145,295	S type	IgG-к type	Bortezomib, dexamethasone	China	2024	0.93	Yes

IgA = immunoglobulin A, IgD = immunoglobulin D, IgG = immunoglobulin G, P = pancreatic-type amylase.

Some studies have suggested that elevated serum amylase may serve as a useful prognostic marker in cases of MM.^[12,29–31]^ Ross et al^[18]^ demonstrated that in patients with Bence Jones protein-type MM, serum amylase levels decreased following treatment and increased upon relapse, with changes in amylase levels reflecting treatment efficacy and disease progression. Massimiliano et al^[21]^ reported a case of a 79-year-old Italian male with lambda-light chain MM who exhibited salivary-type (S) hyperamylasemia during the course of the disease. The patient’s serum amylase progressively increased as the disease advanced, and he died 16 months after diagnosis. Similarly, Calvo-Villas et al^[32]^ described a 71-year-old female MM patient in whom hyperamylasemia was the first abnormal indicator detected prior to her MM diagnosis. As the disease progressed, changes in serum amylase levels paralleled those of other markers of myeloma activity. These findings align with the observations of Ross et al, suggesting that serum amylase may be a useful marker for monitoring disease progression and treatment response in MM. In the present case, the patient’s survival was too short to observe the relationship between serum amylase levels and MM treatment efficacy, as the patient died on the fifth day following the first cycle of chemotherapy. Malignancy-associated serum amylase levels are generally >1000 U/L.^[33]^ At diagnosis, this patient’s serum amylase level was 145,295 U/L, and it remained significantly high at 58,799 U/L prior to chemotherapy. Persistent, markedly elevated serum amylase levels suggest poor prognosis and high mortality risk, indicating that sustained hyperamylasemia may be a marker of aggressive disease progression in MM.

Hata et al^[34]^ reported a case of IgA-λ-type MM in which malignant myeloma cells from the patient’s pleural effusion were cultured. Amylase activity in the supernatant of the cultured myeloma cells showed a linear increase from the initiation of cell culture until cell death. Additionally, cytochemical and immunoelectron microscopy confirmed the presence of both IgA and salivary-type (S) amylase within the myeloma cells, suggesting ectopic amylase production by myeloma cells. Some researchers have even established amylase-producing myeloma cell lines. In 1988, Japanese researchers Matsuzaki et al^[29]^ first proposed non-epithelial tumor cell lines and established 2 human myeloma cell lines, KHM-1A and KHM-1B, from a 54-year-old male patient with IgA-type MM. KHM-1A cells secreted only IgA, while KHM-1B cells secreted both IgA and S-type amylase. In 1989, Ohtsuki et al^[35]^ derived 2 human myeloma cell lines, KMS-12-PE and KMS-12-BM, from a 64-year-old female patient with nonsecretory MM. The KMS-12-PE cell line, originating from pleural effusion, produced ectopic S-type amylase, whereas the KMS-12-BM cell line, derived from bone marrow, did not. However, the exact mechanism of S-type amylase production by malignant plasma cells remains unclear. Fujii et al^[12]^ reported the presence of amylase-containing secretory granules in many epithelial tumor cells but failed to detect such granules in myeloma cells. Nomura et al observed that in amylase-producing cancer cell lines, the zymogen granules disappeared after prolonged culture, yet amylase secretion persisted. Several studies have also been unable to demonstrate the presence of secretory granules. The first autopsy case of IgA-λ-type MM producing S-type amylase was described in Japan. In amylase-producing KHM-1B cells, electron microscopy revealed well-developed endoplasmic reticulum, and immunoelectron microscopy demonstrated reaction products of amylase and immunoglobulin chains within the endoplasmic reticulum. This suggests that myeloma cells may directly secrete amylase via the endoplasmic reticulum, similar to the secretion mechanism of immunoglobulins.^[36]^ One hypothesis attributes this phenomenon to structural alterations and rearrangements in genes near the amylase loci on chromosome 1, including deletions, mutations, or translocations.^[14,30,37]^ Chromosomal translocations involving 1p13 or 21 (near the amylase gene locus) and 9q34 (oncogene locus) have been proposed, indicating that amylase genes might be activated by oncogenes. A case reported by British researchers involved a patient with lambda-light chain MM who developed S-type hyperamylasemia during disease progression. This change was believed to induce plasma cells to produce uncontrolled amounts of amylase, leading to elevated serum amylase levels.^[21]^ However, the precise relationship between chromosomal alterations, amylase production, and malignancies remains unclear. Nair et al^[16]^ described a 47-year-old African American woman diagnosed with MM who, despite metastatic tumors, did not initially present with hyperamylasemia. Following chest wall radiation therapy, elevated amylase levels were observed. This suggests that radiation therapy may have induced mutations in chromosome 1, triggering structural changes in tumor cells and increased amylase production. In conclusion, the mechanisms underlying amylase production in MM are complex and require further investigation.

This case highlights 3 imperatives: investigate malignancy in hyperamylasemia with normal lipase; expedite neuroimaging for new-onset diplopia with systemic symptoms in elderly patients; and recognize salivary hyperamylasemia as a potential marker of aggressive myeloma with extramedullary spread. Vigilance for these presentations enables earlier diagnosis.

## 5. Limitation

A key limitation is the lack of immunohistochemical or molecular confirmation of amylase production by malignant plasma cells. While clinical and biochemical evidence strongly supports the diagnosis, the cellular source and genetic mechanisms remain unconfirmed due to the patient’s rapid deterioration and palliative care direction.

## 6. Conclusion

MM with concurrent hyperamylasemia is a rare condition. By systematically analyzing the patient’s atypical clinical presentation and abnormal laboratory findings, a definitive diagnosis was ultimately achieved. Compared to typical MM patients, those with amylase-producing myeloma tend to have larger tumor burdens, more extensive extramedullary dissemination, greater bone destruction, and significantly shorter survival times. Binocular diplopia may represent a clinical manifestation of extramedullary metastasis in MM. Clinicians should remain vigilant for malignancies in cases of elevated amylase levels after excluding pancreatitis and parotitis, to avoid missed or misdiagnoses. Prompt diagnosis and early initiation of treatment are critical for improving patient survival. Further investigation is required to elucidate the mechanisms underlying hyperamylasemia in MM.

## Acknowledgments

The authors thank the patient’s family for their consent and support, and acknowledge the clinical team for their expert care.

## Author contributions

**Data curation:** Xiaohui Zhang.

**Formal analysis:** Renhua Fan.

**Investigation:** Xiaohui Zhang, Weidong Zhou, Renhua Fan.

**Methodology:** Xiaohui Zhang.

**Supervision:** Weidong Zhou, Renhua Fan.

**Writing – original draft:** Xiaohui Zhang.

**Writing – review & editing:** Weidong Zhou.

## References

[1] Dimopoulos MA Moreau P Terpos E. Multiple myeloma: EHA-ESMO Clinical Practice Guidelines for diagnosis, treatment and follow-up. Ann Oncol. 2021;32:309–22. 33549387 10.1016/j.annonc.2020.11.014

[2] Malard F Neri P Bahlis NJ. Multiple myeloma. Nat Rev Dis Primers. 2024;10:45. 38937492 10.1038/s41572-024-00529-7

[3] Kyle RA Rajkumar SV. Multiple myeloma. N Engl J Med. 2004;351:1860–73. 15509819 10.1056/NEJMra041875

[4] Burtis CA Ashwood ER eds. Tietz Textbook of Clinical Chemistry. 2nd ed. W.B. Saunders; 1994.

[5] Levitt MD Ellis C. A rapid and simple assay to determine if macroamylase is the cause of hyperamylasemia. Gastroenterology. 1982;83:378–82. 6177576

[6] Lopez J Ulibarrena C Gonzalez-Porque P. Amylase-producing Bence Jones multiple myeloma with pancreatitis-like symptoms. Acta Haematol. 1993;90:99–101. 7506860 10.1159/000204384

[7] Reynolds TM. Amylase. Br J Hosp Med (Lond). 2009;70:M8–9. 19357566 10.12968/hmed.2009.70.Sup1.37706

[8] Azzopardi E Lloyd C Teixeira SR Conlan RS Whitaker IS. Clinical applications of amylase: novel perspectives. Surgery. 2016;160:26–37. 27117578 10.1016/j.surg.2016.01.005

[9] Kanno M Ohtake S Okafuji K. IgD-type multiple myeloma associated with tumor of the pancreas. Rinsho Ketsueki. 1988;29:2312–6. 2469811

[10] Wada H Ohtsuki T Sugihara T. Ectopic production of amylase by a non-producing type of multiple myeloma. Rinsho Ketsueki. 1990;31:1022–7. 1699005

[11] Tagawa S Doi S Taniwaki M. Amylase-producing plasmacytoma cell lines, AD3 and FR4, with der(14)t(8;14) and dic(8)t(1;8) established from ascites. Leukemia. 1990;4:600–5. 1697013

[12] Fujii H Yashige H Kanoh T Urata Y. Amylase-producing multiple myeloma. Arch Pathol Lab Med. 1991;115:952–6. 1718241

[13] Hirota K Waga K Nagashima S. Amylase-producing plasma cell dyscrasia. Genomic analysis in a case of intramuscular tumor formation and a review of the literature. Rinsho Ketsueki. 1993;34:177–82. 7684094

[14] Yamakawa T Nagoshi H Takahashi A. Acquired amylase production induced by radiotherapy in a myeloma patient. Rinsho Ketsueki. 1995;36:1175–81. 8531327

[15] Guo MD Ding J Chen YP Wang QY Weng X Ye XH. Multiple myeloma with hyperamylasaemia: a case report and literature review. Chin Gen Pract. 2019;22:1372–6.

[16] Nair S Sachan P Hertan H Pitchumoni CS. Metastatic multiple myeloma with hyperamylasaemia and hyperlipasaemia. Postgrad Med J. 1998;74:621–3. 10211365 10.1136/pgmj.74.876.621 PMC2361001

[17] Weiss MJ Edmondson HA Wertman M. Elevated serum amylase associated with bronchogenic carcinoma; report of case. Am J Clin Pathol. 1951;21:1057–61. 14885124 10.1093/ajcp/21.11.1057

[18] Ross CM Devgun MS Gunn IR. Hyperamylasaemia and multiple myeloma. Ann Clin Biochem. 2002;39:616–20. 12564849 10.1177/000456320203900615

[19] Gomi K Kameya T Tsumuraya M. Ultrastructural, histochemical, and biochemical studies of two cases with amylase, ACTH, and beta-MSH producing tumor. Cancer. 1976;38:1645–54. 186172 10.1002/1097-0142(197610)38:4<1645::aid-cncr2820380434>3.0.co;2-j

[20] Moriyama T. Sialyl salivary-type amylase associated with ovarian cancer. Clin Chim Acta. 2008;391:106–11. 18294455 10.1016/j.cca.2008.01.025

[21] Pinelli M Bindi M Rosada J Scatena P Castiglioni M. Amylase: a disease activity index in multiple myeloma? Leuk Lymphoma. 2006;47:151–4. 16321841 10.1080/10428190500262144

[22] Moriyama T Tozawa T Nobuoka M Ikeda H. Sialyl salivary-type amylasemia associated with immunoglobulin D-type multiple myeloma. Clin Chim Acta. 1995;233:127–34. 7538921 10.1016/0009-8981(94)05971-t

[23] Sagristani M Guariglia R Pocali B. Macroamylasemia in a patient with multiple myeloma. Leuk Lymphoma. 2002;43:1705–7. 12400618 10.1080/1042819021000003081

[24] Shigemura M Moriyama T Endo T. Myeloma cells produce sialyl salivary-type amylase. Clin Chem Lab Med. 2004;42:677–80. 15259386 10.1515/CCLM.2004.115

[25] Sosnoff DR Friend RB Berkovic M Rasansky RJ Hoffman SM. Salivary amylase-producing multiple myeloma: case report and review of the current literature. J Clin Oncol. 2013;31:e309–11. 23690421 10.1200/JCO.2012.46.4677

[26] Bloemendal HJ Lobatto S. Hyperamylasaemia in multiple myeloma. Neth J Med. 1996;49:38–41. 8772359 10.1016/0300-2977(95)00110-7

[27] Moriyama T Tozawa T Yamashita H Onodera S Nobuoka M Makino M. Separation and characterization of sialic acid-containing salivary-type amylase from patients’ sera with immunoglobulin A-type myeloma. J Chromatogr. 1991;571:61–72. 1725778 10.1016/0378-4347(91)80434-e

[28] Li SX Wang XL Cong MQ Yin YJ. Analysis of a case with multiple myeloma and hyperamylasemia in a plateau region. J Pract Clin Med. 2024;28:103–6.

[29] Matsuzaki H Hata H Takeya M Takatsuki K. Establishment and characterization of an amylase-producing human myeloma cell line. Blood. 1988;72:978–82. 2458153

[30] Hata H Matsuzaki H Sanada I Takatsuki K. Genetic analysis of amylase-producing cell lines: ectopic activation of the amylase gene by translocation. Jpn J Clin Oncol. 1990;20:246–51. 1701504

[31] Lee JW Lee JK Hong YJ Hong SI Chang YH. Correlation of chromosomal aberrations with prognostic markers in multiple myeloma patients--a single institution study. Korean J Lab Med. 2008;28:413–8. 19127104 10.3343/kjlm.2008.28.6.413

[32] Calvo-Villas JM Alvarez I Carretera E Espinosa J Sicilia F. Paraneoplastic hyperamylasaemia in association with multiple myeloma. Acta Haematol. 2007;117:242–5. 17377372 10.1159/000100931

[33] Wu D Liu XH. Chronic hyperamylasemia. Chin J Pract Intern Med. 2007;27:1552–3.

[34] Hata H Matsuzaki H Tanaka K. Ectopic production of salivary-type amylase by a IgA-lambda-type multiple myeloma. Cancer. 1988;62:1511–5. 2458822 10.1002/1097-0142(19881015)62:8<1511::aid-cncr2820620811>3.0.co;2-l

[35] Ohtsuki T Yawata Y Wada H Sugihara T Mori M Namba M. Two human myeloma cell lines, amylase-producing KMS-12-PE and amylase-non-producing KMS-12-BM, were established from a patient, having the same chromosome marker, t(11;14)(q13;q32). Br J Haematol. 1989;73:199–204. 2479409 10.1111/j.1365-2141.1989.tb00252.x

[36] Takeya M Matsuzaki H Hata H Takatsuki K Takahashi K. Amylase-producing multiple myeloma. Cytochemical, immunohistochemical and immunoelectron microscopic studies. Virchows Arch A Pathol Anat Histopathol. 1989;415:219–24. 2474886 10.1007/BF00724908

[37] Delannoy A Hamels J Mecucci C. Amylase-producing IgD-type multiple myeloma. J Intern Med. 1992;232:457–60. 1280672 10.1111/j.1365-2796.1992.tb00615.x

